# ‘I’m fishing really’ — inflammatory marker testing in primary care: a qualitative study

**DOI:** 10.3399/bjgp16X683857

**Published:** 2016-02-08

**Authors:** Jessica Watson, Isabel de Salis, Willie Hamilton, Chris Salisbury

**Affiliations:** Professor in primary health care, Centre for Academic Primary Care, School of Social and Community Medicine, University of Bristol, Bristol.; Professor in primary health care, Centre for Academic Primary Care, School of Social and Community Medicine, University of Bristol, Bristol.; Professor of primary care diagnostics, University of Exeter Medical School, Exeter.; Professor in primary health care, Centre for Academic Primary Care, School of Social and Community Medicine, University of Bristol, Bristol.

**Keywords:** acute-phase proteins, C-reactive protein, diagnosis, general practice, primary health care, qualitative research

## Abstract

**Background:**

Inflammatory markers can be helpful as part of the diagnostic workup for specific diseases or for monitoring disease activity. A third use is as a screening and/or triage tool to differentiate between the presence or absence of disease. Most research into inflammatory markers looks at diagnosis of specific diseases and comes from secondary care. Qualitative studies to explore when and why clinicians use these tests in primary care are lacking.

**Aim:**

To identify clinicians**’** approaches to inflammatory marker testing in primary care.

**Design and setting:**

Qualitative study with 26 GPs and nurse practitioners.

**Method:**

Interviews were conducted using a semi-structured topic guide. Clinicians reviewed recent cases of inflammatory marker testing in their pathology inbox. Interviews were audiorecorded and transcribed. Qualitative analysis was conducted by two of the authors.

**Results:**

Clinicians are uncertain about the appropriate use of inflammatory markers and differ in their approach to testing patients with undifferentiated symptoms. Normal or significantly elevated inflammatory markers are seen as helpful, but mildly raised inflammatory markers in the context of non-specific symptoms are difficult to interpret. Clinicians describe a tension between not wanting to **‘**miss anything**’** and, on the other hand, being wary of picking up borderline abnormalities that can lead to cascades of further tests. Diagnostic uncertainty is a common reason for inflammatory marker testing, with the aim to reassure; however, paradoxically, inconclusive results can generate a cycle of uncertainty and anxiety.

**Conclusion:**

Further research is needed to define when inflammatory marker testing is useful in primary care and how to interpret results.

## INTRODUCTION

Inflammatory markers including C-reactive protein (CRP), plasma viscosity (PV), and erythrocyte sedimentation rate (ESR) are long established tools for detection and monitoring of a variety of inflammatory conditions. Scandinavian studies in the 1990s suggested inflammatory markers are measured in approximately 4% of GP consultations, for a range of indications, with 44–47% for specific diagnostic purposes, 27–33% for monitoring disease, and 14–28% for non-specific diagnostic purposes.^1^,^2^ Testing rates have since significantly increased: CRP testing in the UK rising by 85.8% between 2005 and 2009.^3^ Large regional variations in testing rates have been noted, particularly with regard to PV.^3^ This could reflect different regional policies, but may indicate that GPs are uncertain about the appropriate use of these tests. There is also considerable inter-practice variation in GPs’ responses to abnormal inflammatory markers.^2^,^4^,^5^ Total costs of testing must be considerable; for example, over 120 000 primary care requests for inflammatory markers were processed in 2014 at North Bristol NHS Trust, costing £177 000, for a population of 500 000 (P Virgo, personal communication, 2015). In 2006 overall annual pathology testing was estimated at £2.5 billion, nearly 4% of NHS expenditure.^6^

The classic conditions for which inflammatory markers are recommended as first line are polymyalgia rheumatica (PMR), giant cell arteritis,^7^,^8^ and myeloma.^9^ Systematic reviews have also assessed the utility of CRP in diagnosing appendicitis,^10^ neutropenic sepsis^11^,^12^ and serious infection in febrile children,^13^,^14^ joint infection,^15^,^16^ chorioamnionitis,^17^ and several cancers in adults.^18^–^23^ These studies are mostly based in secondary care, so may be less applicable to primary care’s low disease prevalence settings. Primary care studies have demonstrated that point-of-care CRP testing for diagnosis of lower respiratory tract infections can reduce antibiotic prescribing and enhance patients’ and GPs’ confidence in prescribing decisions;^24^–^27^ this is now incorporated into the National Institute for Health and Care Excellence (NICE) pneumonia guidelines.^28^

Another use of inflammatory markers is as a general indicator to differentiate between the presence and absence of disease. Evidence for this is based on small, old studies, mostly looking at ESR, which is now little used in clinical practice.^29^–^32^

When and why clinicians use inflammatory markers in primary care, including benefits and pitfalls of testing, have been little explored and qualitative studies are lacking. This study aimed to understand the real-life complexities of test use from a social science perspective, through qualitative interviews with GPs and nurse practitioners who order these tests.

**How this fits in**

Inflammatory markers are used in primary care as part of the diagnostic workup for specific diseases or for monitoring disease activity. Clinicians also use them as a non-specific marker to differentiate between the presence or absence of disease in cases of diagnostic uncertainty. Interpretation of abnormal results in this context can be difficult and can lead to increased uncertainty and a cascade of further tests. Further research in primary care is needed to guide clinicians on when to test inflammatory markers and how to interpret abnormal results.

## METHOD

### Recruitment

Thirty research-active primary care practices from a total of 55 practices in the Bristol Clinical Commissioning Group were invited by e-mail to participate via the e-bulletin of the West of England Clinical Research Network. Expressions of interest were received from 14 practices; of these 10 participated. Purposive sampling was used to ensure a diversity of participants in terms of sex, years qualified, and practice role. In total 26 GPs and nurse practitioners were interviewed.

### Interviews

Interviews were carried out by one of the authors and took place in participants’ GP practices. Participants were told that the interviewer is a GP registrar with an interest in test use in general practice. The interviewer had received training in qualitative research and was supervised by an experienced qualitative researcher. The first two interviews were treated as pilots but as data collection was successful they were subsequently added to the final dataset. Clinicians were paid £60 for participating. Ethical approval was obtained. Interviews were face to face, loosely structured around a topic guide with relevant areas explored in depth, and continued until data saturation was achieved. After briefly eliciting background information of clinicians’ training, current role, and experience of inflammatory marker testing, clinicians were asked to review their pathology inbox to identify and discuss anonymised recent cases of inflammatory marker testing to ensure the inclusion of real-life practice. A topic guide (Box 1) was used to provide prompts to ensure all areas were covered in each interview if they were not already spontaneously brought up. The topic guide evolved during the course of the study using information emerging in early interviews to develop further questions for exploration in subsequent interviews. The interviewer emphasised that the research process was non-judgemental, and that all interviews were confidential and anonymised, without names or practices linked to the comments. All interviews were audiorecorded using an encrypted device and lasted between 23–48 minutes, with an average duration of 32 minutes.

Box 1. **Broad areas covered in topic guide**

1. Professional background, current role, experience of inflammatory marker testing.
2. Review of recent cases of inflammatory marker testing using pathology inbox.
3. Prompts (if necessary) around inflammatory marker testing:
  - reasons for testing;
  - frequency of testing;
  - expectations of tests;
  - choice of test/batteries of tests used;
  - interpretation and management of results;
  - communication and explanations to patients;
  - local guidelines or approaches;
  - other factors influencing testing rates
    — fear of litigation, patient attitudes, secondary care, point-of-care testing.

### Analysis

The first two interviews were transcribed verbatim by the interviewer to increase familiarity with the data; thereafter an independent transcription service was used. Transcripts were checked, corrected, and anonymised by the interviewer prior to analysis. Analysis began when the first transcripts were available, so that data collection and analysis were conducted concurrently, with early interviews informing questions for subsequent interviews. Data were read and re-read to aid familiarity. The same researcher who carried out interviews led analysis, using NVivo. Analysis was thematic with key themes being developed and then compared across the dataset.^33^ Two of the authors derived codes from four early transcripts, which were compared and refined in discussion to develop a single coding framework. The coding framework was applied to further transcripts and revised as necessary. Once coded, data were grouped into key categories or ‘themes’, which arose from the data in interaction with the original research questions using a grounded theory approach.^34^

## RESULTS

Table 1 summarises the characteristics of the 26 participants. Twenty-two clinicians were recruited from 10 GP practices; four were locum GPs. Practices included a broad range of urban and suburban practices, with varying levels of socioeconomic deprivation; no rural practices were included. From the data four main themes emerged: reasons for inflammatory marker testing; interpretation and management of raised inflammatory markers; pitfalls of testing; and clinicians’ self-doubt about testing. Most of the data was elicited during discussions of clinical cases and relatively few prompts were needed.

**Table 1.** Characteristics of participants (*n* = 26)

		**Male**	**Female**
**Role**	GP partner	6	6
	Salaried GP	1	4
	Locum GP	3	1
	Nurse practitioner	–	3
	GP registrar	–	2

**Years experience**	Newly qualified/trainee (first 5 years of qualification)	3	6

	Experienced (>5 years experience)	7	10

### Reasons for inflammatory marker testing

Inflammatory marker testing was performed for three main reason: diagnosis, monitoring, and ‘screening’. In diagnosis, clinicians were often trying to differentiate minor illness from more serious conditions, for example, musculoskeletal joint pains versus inflammatory arthritis. The inflammatory marker alone was rarely diagnostic; with the possible exception of PMR:

‘PMR is a good example. Good story, raised viscosity, you’ve got your diagnosis in the bag and you feel happy doctor, because it’s very rare that anything like that happens.’

(Interview 11, male, locum GP, 16 years’ experience)

Sometimes inflammatory markers were part of the workup for referral, for example, to chronic fatigue clinic, memory clinic, or for patients potentially needing hospital admission:

‘CRP’s good for that, the orthopaedic surgeons respect it and other people do as well. If you want to get somebody in to hospital you say their CRP’s just gone from sort of 10 to 100, oh right, okay, I’ll see them.’

(Interview 2, male, GP partner, 32 years’ experience)

Inflammatory markers were also used for monitoring response to treatment, for example, when reducing steroids in PMR, or at the request of secondary care colleagues:

*‘There’s all the people who are on high-risk drug monitoring, so all the people on methotrexate and all the rest of it, they have them done* ad infinitum *and we don’t fiddle with them because the hospital have said you should have some done.’*

(Interview 7, male, GP partner, 7 years’ experience)

Finally, ‘screening’ was a term used by participants to mean the testing of patients with non-specific or undifferentiated symptoms without a clear diagnosis in mind. This could assist triage, or differentiate between the presence or absence of disease:

‘I’m fishing really. So it’s, a lot of our work is early presentation of undifferentiated disease and I get, essentially buying time I get very strongly reassured, rightly or wrongly, by negative inflammatory markers.’

(Interview 7, male, GP partner, 7 years’ experience)

A clinician’s intuition of serious underlying pathology could trigger such use:

‘Occasionally I may do it in someone where I’ve just got a slightly bad feeling about them, can’t put my finger on it but I’m kind of thinking, I do an inflammatory marker because it might help me potentially rule out, you know any nasty cancer or something that’s going on that I can’t quite put my finger on and if it comes up positive I’ll dig a little deeper, but if it comes back negative then I will be more comfortable with watching and waiting.’

(Interview 11, male, locum GP, 16 years’ experience)

Clinicians talked about a fear of *‘missing something’*, especially something serious such as cancer, and used inflammatory markers to manage diagnostic uncertainty and to reassure themselves there was *‘nothing serious going on’*. Clinicians often expected normal results and used the test to help *‘rule out’* serious pathology:

‘So if we had a test that, a single blood test, that doctors could do which would reassure the patient there was nothing bloody wrong at all, then that would be a very popular test. We’ll have the “nothing wrong at all” test for you, sir … You know, all the other tests are, well, you might have this specifically wrong with you or you might have this … But the CRP is probably the closest thing that we’ve got to a “nothing wrong at all” test.’

(Interview 21, male, locum GP, 25 years’ experience)

However, clinicians’ opinions varied about using inflammatory markers for ‘screening’, with some only using them when they had a specific diagnosis in mind.

### Interpretation and management of raised inflammatory markers

Normal or significantly elevated inflammatory markers were considered helpful and could usually be interpreted in the clinical context. However, mildly raised inflammatory markers in the context of non-specific symptoms were perceived as problematic:

‘So I think that if they’re very normal that’s good, if they’re very abnormal that’s helpful. I think that probably where the problem lies is if you get like a mildly raised viscosity and you wonder is that, how significant is that? And then if you requested it you’ve then got that and then what do you do about it …?’

(Interview 23, female, salaried GP, 1 year’s experience)

Most clinicians repeated a raised inflammatory marker result and then considered further additional tests, but were uncertain and differed about how long to wait and which tests to perform next. Compared with other blood tests clinicians felt this was a particular challenge with inflammatory markers due to their non-specific nature. Very few cases of inflammatory markers revealed unexpected pathology that would otherwise have been missed. Rare examples were an unexpected case of tuberculosis and a retropharangeal abscess in someone who *‘just looked ill’*; both had significantly elevated inflammatory markers. In contrast, cascades of further tests were frequent following a raised inflammatory marker in a hunt for pathology likened to ‘looking for a needle in a haystack’. If no cause could be found patients were sometimes referred to secondary care:

‘Then you think suddenly, well should I be looking further and further and further, but that could mean more and more random investigations until you get the point where you goes, oh, I’ll just do a whole body CT scan to see if anything pops up I suppose.’

(Interview 5, male, GP partner, 6 years’ experience)

### Pitfalls with inflammatory marker testing

Clinicians gave several reasons to avoid inflammatory marker testing that stemmed from the non-specific nature of the tests. First, clinicians suggested they would not change management or that the tests were just ‘unhelpful’. Consequently, some emphasised the importance of considering why they were ordering the test and how they would manage the result *before* making the request. Without such careful consideration, borderline results could be difficult to interpret and potentially lead to uncertainty and anxiety. A particular challenge was dealing with a colleague’s blood results without knowledge of the patients’ symptoms and reasons for testing:

‘I sometimes think I shouldn’t have done that test because it’s just complicated things.’

(Interview 3, female, GP partner, 20 years’ experience)

Interpreting abnormal results could also be difficult in patients with other potential reasons for raised inflammatory markers, for example, older patients and patients with comorbidities or obesity:

‘So well they’re surprisingly unhelpful in some ways, aren’t they? Over the years I’ve learned that very old people usually have a plasma viscosity that’s a little bit up … so you ignore the mildly raised viscosity in the older person and so you … have to be more careful not to do it if it’s not going to influence your management. And the other people who have a raised plasma viscosity are obese people and maybe the people with high cholesterol as well.’

(Interview 9, female, partner, 23 years’ experience)

Finally, clinicians noted the workload generated by inflammatory marker testing, in particular with borderline results generating uncertainty and requiring further action:

‘Well, we were coming up with lots of mildly abnormal results, so mildly raised, and what does that mean? Is that okay? Can we live with that? Do we then have to repeat the test? … I kind of realised that I was becoming besieged by these PV results, which were, I didn’t really know what to do with.’

(Interview 22, female, salaried GP, 10 years’ experience)

### Clinicians’ self-doubt

Clinicians expressed uncertainty about whether they were doing the *‘right thing’* and felt it was an area that lacked teaching and evidence-based guidance. There was considerable variation in which inflammatory marker to use according to clinicians’ training and experience. In general, clinicians considered plasma viscosity better for ‘screening’ and CRP more useful for diagnosis or monitoring of infections or specific inflammatory conditions. Some used both CRP and viscosity but felt they were *‘probably not supposed to’*:

‘Having had this discussion it’s kind of left me with questions and definitely thinking should I, shouldn’t I be doing that? ‘

(Interview 25, female, salaried GP, 7 years’ experience)

‘With GPs, a lot of things are oh well it is what I do and it’s what I have always done and erm it seems to work, so yeah I’ll just do it a bit more … so what we end up doing is kind of making it up, so if you have got somebody who has got slightly abnormal CRP, when do you re-check it? There’s no guidelines, so you think to yourself well you know perhaps it will be 2 weeks or maybe 4 weeks or maybe when their chest infection has gone?’

(Interview 19, female, GP partner, 13 years’ experience)

Nurse practitioners used inflammatory markers less frequently, both because they tended to see minor illnesses and because they held a perception that inflammatory markers were *‘a bit out of my sphere of competence’* unless with GP guidance. Consequently, they had fewer concerns about the uncertainty of test results and management of abnormal results could be *‘handed over’* to GP colleagues for follow-up.

### Cycle of uncertainty and anxiety with inflammatory marker testing

**Figure 1.**
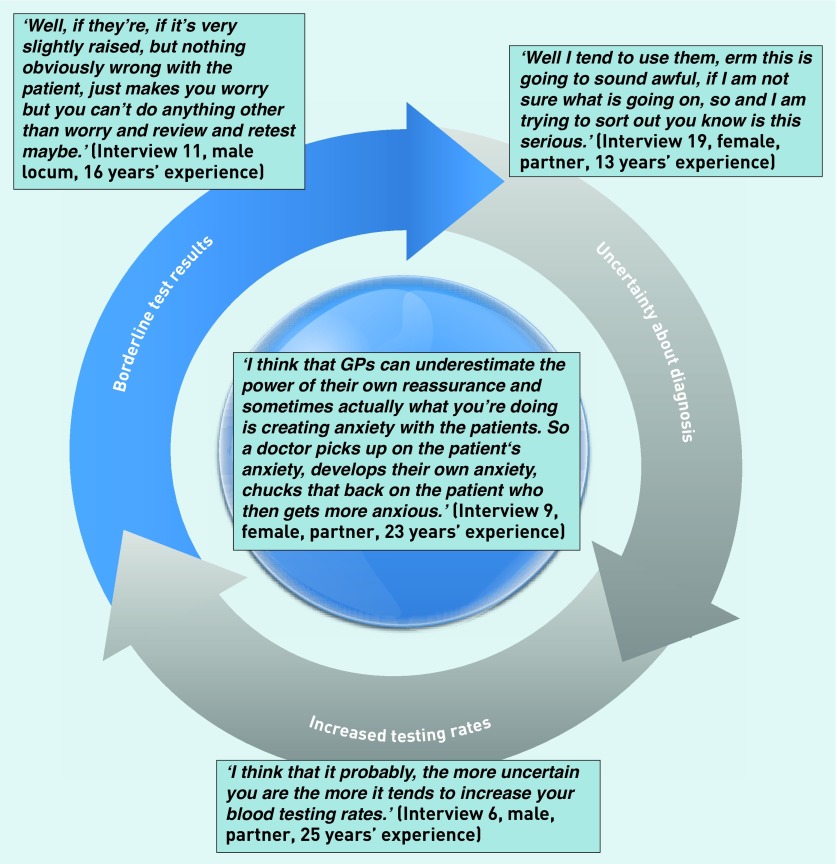
Cycle of uncertainty and anxiety in inflammatory marker testing. Uncertainty about diagnosis can lead to increased testing rates. This increases the chance of borderline test results, which may cause more uncertainty and anxiety.

As demonstrated, diagnostic uncertainty was a common reason for inflammatory marker testing, as a ‘screening’ tool for serious disease. Although the aim of testing was often *‘for reassurance’*, inconclusive or borderline results could paradoxically generate increased uncertainty and anxiety (Figure 1). This led to a tension between clinicians wanting to make sure they were not *‘missing something’*, and conversely not wanting to generate unhelpful or uninterpretable results. This tension was particularly apparent with inflammatory markers compared with other blood tests:

‘I think there is a bit of a mystique about the usefulness of CRP testing, and we all know what an FBC and U&Es, and it’s very well defined, isn’t it? But the implications and the usage of CRP is very vague, it’s a kind of, no one ever taught us about the practicalities of ordering a CRP, and that’s one of the issues really.’

(Interview 4, male, GP partner, 17 years’ experience)

## DISCUSSION

### Summary

As far as we are aware this is the first qualitative study into the use of inflammatory markers in primary care. Clinicians used inflammatory markers for diagnosis and monitoring, yet differed particularly in their approach to ‘screening’ patients with undifferentiated symptoms. Normal or significantly raised inflammatory markers were considered helpful but borderline abnormalities were difficult to interpret due to the non-specific nature of the tests. Clinicians described a tension between not wanting to ‘miss anything’ and, on the other hand, being wary of picking up borderline abnormalities leading to further tests. This tension was compounded by clinicians’ uncertainty about whether they were doing the ‘right thing’. Diagnostic uncertainty was a common reason for inflammatory marker testing aiming to reassure; however, paradoxically, inconclusive results could generate a cycle of increased uncertainty and anxiety.

This difficulty interpreting abnormal results and the consequent uncertainty is a particular characteristic of inflammatory markers due to their non-specific nature; however, it is unlikely to be a unique problem. Similar difficulties of interpreting borderline abnormalities could be of relevance for a range of blood tests.

### Strengths and limitations

A strength of this study was the use of real-life cases, which ensured data were grounded in daily clinical practice, thereby reducing the chances of cognitive biases or doctors presenting an idealised practice of what they think they *‘should’* do. Interviews were carried out by a GP registrar, allowing interviewees to feel comfortable discussing cases with a fellow clinician with shared understanding. However, this could influence the reflexivity of the interviewer who had their own a priori experiences of inflammatory marker testing and an interest in the uncertainty and lack of evidence-based guidelines in this area. Although it was emphasised that interviews were non-judgemental, some clinicians may have felt uncomfortable discussing areas of clinical practice where they felt uncertain, defensive, or deficient, and may therefore have reinterpreted their diagnostic argument for testing from intuitive to more rational thoughts. Similarly, clinicians who were less confident about inflammatory marker testing may have declined to take part. This could imply that ‘real-life’ testing is more haphazard and less logical than presented here. Fewer nurse practitioners were sampled than doctors because it became evident in the study that they rarely requested inflammatory markers or managed results. No rural practices participated, where clinicians may have slightly different approaches to blood testing, for example, if laboratory services are less accessible. Interviews were only undertaken with clinicians; in future work, patients’ perspectives would be useful.

### Comparison with existing literature

Despite little evidence supporting the use of inflammatory markers for screening or triage^35^ it was observed that many GPs use these tests for patients with undifferentiated symptoms. This mirrors research among Dutch GPs reporting that unexplained complaints are positively associated with test ordering behaviour (odds ratio 2.4, 95% confidence interval = 1.2 to 5.3).^36^ Patients with unexplained complaints presenting with fatigue and longer duration of symptoms are more likely to have blood tests done.^37^

Some GPs avoided inflammatory markers for non-specific purposes in case results were unhelpful. This concurs with previous research on ESR showing that generally, when GPs use inflammatory markers for non-specific purposes, results are afterwards seen as being of little or no clinical value.^1^ Previous questionnaire studies looking at blood testing generally have shown that abnormal test results are common, even when tests are requested for reassurance or to exclude disease with low pre-test probability, suggesting GPs should be cautious about using blood tests for ‘screening’ or non-specific purposes.^38^ Other studies focusing on tests in general have shown that, although doctors may be reassured by negative testing when no disease is suspected,^39^ normal diagnostic tests make little difference to the level of patient reassurance.^40^,^41^ Furthermore, this study has shown that borderline results may actually increase anxiety, rather than reassure, and lead to further testing, a pitfall which Deyo called the cascade effect of medical technology.^42^ Sah and colleagues explored this issue of inconclusive tests, coining the term *‘*‘investigation momentum’: the concept that the psychological uncertainty experienced after an inconclusive test leads to additional testing in the *‘relentless pursuit to resolve uncertainty’*^43^ Although some clinicians in this study perceived that abnormal test results could increase patient anxiety and lead to further cascade testing, the frequency of ‘cascade testing’ and patients’ views are under-researched.

### Implications for research and practice

This study highlights a problem with inflammatory marker testing, which may ring true for practising clinicians but is rarely discussed in research literature. Clinicians should consider the potential pitfalls of inflammatory marker testing and think about how they will use the results before requesting the tests. Estimates from a UK Department of Health-commissioned review of pathology services in 2008 calculated that approximately 25% of pathology tests were unnecessary, representing considerable waste.^6^ An Academy of Medical Royal Colleges report has called for doctors to take responsibility for cutting waste in a finite system, with overuse of diagnostic tests being one of three core areas of suggested focus.^44^ More recently, the potential harm to patients has also been highlighted, with the initiative ‘Choosing Wisely’^45^ aiming to identify areas not supported by evidence, not free from harm, or not truly necessary.

Inflammatory markers, performed without clear clinical indication, may potentially be such an unnecessary intervention. However, the opposite problem, of under-testing and missed diagnosis, is also a concern, and in fact both may coexist. In particular, much of the UK’s poor record in cancer outcomes is blamed on diagnostic delays. One factor that has received little attention is raised inflammatory markers that have been shown to be of relevance in breast,^18^,^19^ lung,^20^ colon,^21^ ovarian,^22^ and urological cancers.^23^ Further research is needed to help clinicians interpret raised inflammatory markers, as previous studies usually start with a single disease state, often in secondary care settings, and estimate the probability of raised inflammatory markers. However, clinicians start with a test result and need to know the predictive probability of a wide range of possible diseases, including risk of cancer. Research is also needed to quantify the benefits and harms of using inflammatory markers for patients with undifferentiated symptoms as a tool to try to ‘rule out’ serious pathology.
